# Modeling tumor cell adaptations to hypoxia in multicellular tumor spheroids

**DOI:** 10.1186/s13046-017-0570-9

**Published:** 2017-08-03

**Authors:** Stephen Riffle, Rashmi S. Hegde

**Affiliations:** Division of Developmental Biology, Cincinnati Children’s Hospital Medical Center, University of Cincinnati College of Medicine, 3333 Burnet Avenue, Cincinnati, OH 45229 USA

**Keywords:** Hypoxia, Multicellular Tumor Spheroids, Metabolism, DNA Damage Repair, Proliferation, Cancer

## Abstract

Under hypoxic conditions, tumor cells undergo a series of adaptations that promote evolution of a more aggressive tumor phenotype including the activation of DNA damage repair proteins, altered metabolism, and decreased proliferation. Together these changes mitigate the negative impact of oxygen deprivation and allow preservation of genomic integrity and proliferative capacity, thus contributing to tumor growth and metastasis. As a result the presence of a hypoxic microenvironment is considered a negative clinical feature of many solid tumors. Hypoxic niches in tumors also represent a therapeutically privileged environment in which chemo- and radiation therapy is less effective. Although the negative impact of tumor hypoxia has been well established, the precise effect of oxygen deprivation on tumor cell behavior, and the molecular signals that allow a tumor cell to survive in vivo are poorly understood. Multicellular tumor spheroids (MCTS) have been used as an in vitro model for the avascular tumor niche, capable of more accurately recreating tumor genomic profiles and predicting therapeutic response. However, relatively few studies have used MCTS to study the molecular mechanisms driving tumor cell adaptations within the hypoxic tumor environment. Here we will review what is known about cell proliferation, DNA damage repair, and metabolic pathways as modeled in MCTS in comparison to observations made in solid tumors. A more precise definition of the cell populations present within 3D tumor models in vitro could better inform our understanding of the heterogeneity within tumors as well as provide a more representative platform for the testing of therapeutic strategies.

## Background

The majority of solid tumors will develop hypoxia to some degree and tumor hypoxia is a significant prognostic factor that predicts poor patient outcome [1, 2]. It is clear from decades of research that hypoxia induces metastasis and invasion, imparts chemo- and radiation resistance, and provides a selective pressure to abrogate pro-apoptotic signaling [3]. The clinically relevant nature of hypoxia has prompted investigations into how the tumor microenvironment directs tumor cell biology and function. Although the literature on this topic is extensive [1–7], many aspects of tumor cell biology and survival in the context of a 3-dimensional (3D) environment remain poorly understood. For decades the Multicellular Tumor Spheroid (MCTS) model has been used to study clinically relevant aspects of tumor biology, including hypoxia [8], protein expression patterns within tumors [9–11], and responses to therapeutics [9, 10, 12–23]. However, relatively few experiments have attempted to use MCTS to further our understanding of tumor cell adaptations within a hypoxic microenvironment. This review aims to describe ways in which MCTS can be used to better simulate solid tumors by detailing key features of MCTS that resemble the in vivo context.

### The development of tumor hypoxia

While the term hypoxia is used to describe a wide variety of oxygen concentrations [2, 7], it most often refers to the point at which oxygen concentrations have decreased beyond the threshold required for normal cell function. The majority of solid tumors will develop hypoxic regions due to a combination of rapid oxygen depletion, insufficient vascularization, and suboptimal tumor blood flow [2, 7]. For example, the consumption of oxygen by rapidly proliferating perivascular tumor cells can deplete the limited supply of available oxygen and prevent sufficient oxygenation of subsequent cell layers [8, 24–26]. While intracellular oxygen is utilized in a variety of reactions, the majority of oxygen consumption is devoted to ATP production through glucose metabolism [26, 27] where oxygen serves as a terminal electron receptor during oxidative phosphorylation. In addition to consumption through intracellular processes, the physical distance between tumor cells and blood vessels also influences the development of hypoxia. Oxygen diffusion through tissue is limited to approximately 200 μm based on evidence from experimental and mathematical models [3, 28]. Hypoxia can be further exacerbated by the destruction of angiogenic vessels following cytotoxic or anti-angiogenic therapy [8, 29–31]. Accumulating evidence now suggests that antiangiogenic therapy induces tumor hypoxia which provides a selective pressure for tumors to acquire a more aggressive phenotype leading to therapeutic resistance and tumor progression [29–31]. Whether developed as a result of rapid tumor growth or in response to therapeutics, hypoxia is ultimately the result of an imbalance between oxygen availability, consumption, and the physical boundaries to oxygen diffusion inherent to a 3D tissue mass.

### Spheroid models for studying hypoxia

The effect of hypoxia on cells has traditionally been studied in monolayer culture. 2D (monolayer) hypoxia experiments are most typically performed by placing tumor cells in a gas-controlled chamber [2]. While experimentally straightforward, this method is unable to recreate clinically relevant aspects of tumor biology that can impact on tumor cell behavior and therapeutic response [13, 32]. For example, monolayer cells experience polarized cell adhesion and two dimensional contact with neighboring cells which results in abnormal cell spreading, alterations in the distribution of cell surface receptors, and selection for specific sub-populations of cells best adapted to in vitro growth [32]. It is also well established that the genomic profiles and therapeutic responses of tumor cells grown in 2D differ from those seen in solid tumors [9–13, 33]. Studying hypoxia in vivo is challenging due to the high degree of variation in oxygen tensions within and amongst tumors, and limited ability to definitively identify regions of chronic versus acute hypoxia [2, 34]. For these reasons, there can be a disconnect between in vitro studies and the complex 3D environment of a tumor. MCTS may contribute to bridging this gap.

Several methods are used for generating spherical 3D cultures and are reviewed extensively elsewhere [8, 32, 35–37]. Common techniques include the liquid overlay technique in which 3D culture is attained by incubating cell suspensions in plates coated with an inert substrate, and the “hanging drop” method wherein cell suspensions are cultured in suspended droplets such that gravity prevents cell attachment and favors cell-cell adhesion [8, 36, 38]. Several factors, including the size of the spheroids, can influence the behavior of MCTS [39]. Spheroid size and media composition dictate the viability and growth kinetics of 3D cultures due to the development of gradients in oxygen and metabolites (discussed below); therefore 3D culture requires careful design of growth conditions and analytical endpoints [39]. Excellent discussions on the practical considerations needed when designing and interpreting data with 3D culture models have been published elsewhere [12, 39].

There are four types of 3D spherical culture models that differ in some important ways (recently reviewed in [36]): MCTS, Tumor Spheres (TS), Tumor Derived Tumor Spheres (TDTS), and Organotypic Multicellular Spheres (OMS). MCTS are formed from established tumor cell lines and grown in standard culture media. MCTS can be composed of mono- or heterotypic cell populations, the latter being the co-culture of tumor cells with other cell types such as macrophages, endothelial cells, and fibroblasts (discussed later in this review). A variation on MCTS culture is the TS model which has been used as a method for growing tumor stem cells in 3D. These spheres are formed from clonal expansion of single cells suspended in non-adherent conditions supplemented with a specific compliment of growth factors in the culture media [36]. Unlike MCTS derived from established cell lines, TS culture represents a selective population of cells known to be aggressive and likely to contribute to tumor regrowth [36, 40]. Accordingly, TS may differ in drug response and growth kinetics relative to MCTS owing to the enrichment of the cancer stem cell population [40]. TDTS are similar to TS however they are formed from partially dissociated tumor tissues [36, 40, 41]. In the TDTS model, primary tumor cells are separated from non-tumor cell types and grown in non-adherent conditions. Relative to MCTS and TS, TDTS have been shown to more accurately recreate tumor growth and gene expression profiles. For example, in breast and colon cancer, TDTS mimic differentiation properties and growth kinetics of the parent tumors more accurately than MCTS from the same tumor background [36, 42]. TDTS therefore provide a suitable model for studying properties of individual tumors. The OMS model utilizes primary, non-dissociated, tumor tissue comprised of all cell types residing within the tissue at the time of excision which provides additional complexity. The presence of stromal cells can adversely affect tumor therapeutic response; therefor the OMS culture method provides a suitable model in which an individual tumor’s therapeutic response can be studied to predict an in vivo response [36, 43, 44].

Each model has distinct advantages and disadvantages (discussed elsewhere [12, 15, 32, 36]) but for maximal control and reproducibility of cell behavior, the MCTS model provides the best coupling of speed with which spheres can be generated, the ability to eliminate influence from non-tumor cell types, and the heterogeneous phenotype of tumor cells incorporated into the sphere. The properties of 3D culture discussed in this review however are applicable to most 3D systems because critical physical properties, such as the development of hypoxia, within the 3D environment are consistent amongst models [12, 32, 39, 45].

### Tumor cell adaptations under hypoxia

Spheroids are a useful in vitro model of avascular tumor spaces. Spheroids exceeding 400 μm in diameter develop a hypoxic core and activate known survival signaling pathways to maintain cell viability. Tumor cells grown as MCTS, TS, TDTS, and OMS display regional heterogeneity in tumor cell proliferation [44, 46, 47], metabolic activity [9–11, 32, 44, 48, 49], and DNA damage repair (DDR) signaling [9, 50–53]. In addition to the influence of the 3D microenvironment, the genetic background of the tumor cells and stromal cell populations also contribute to the specifics of spheroid regionalization [39, 54–56].

**Fig. 1:**
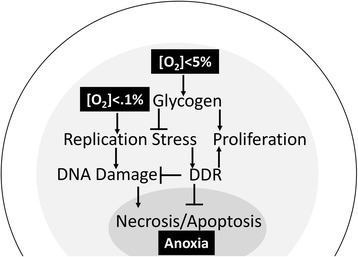
Hypoxia-induced adaptations in a spheroid. Oxygen gradients within tumor spheroids lead to conditions ranging between mild physiological hypoxia to anoxia (represented here by shades of gray). This results in regionalization of tumor cell populations [91, 127]. Hypoxia develops in the spheroid core due to a combination of oxygen diffusion limitations and rapid consumption from proliferating cells [24, 33, 127]. Oxygen deprivation induces glycogen storage to facilitate subsequent metabolism and continued proliferation under more severe hypoxia [49, 62, 71]. Cycling cells in hypoxic regions experience replication stress (stalled replication forks [77] and DNA damage [71]), inducing activation of DNA damage repair (DDR) signaling. DDR allows temporary tumor cell survival and proliferation upon re-oxygenation, but after prolonged periods of severe hypoxia the replisome will disassemble [65, 84]

Hypoxia in the 3D environment of tumors and MCTS induces the stable expression of hypoxia inducible factors (HIF). In vivo, HIF proteins transcriptionally promote angiogenesis through VEGF-A, glycolysis, and pH control through CA-IX [57]. Expression of HIF target genes occurs in tumor regions distal to blood vessels and is commonly used to identify hypoxic conditions [2]. Similar patterns have been observed in MCTS wherein HIF target genes (CA-IX, Glut1, VEGF-A) are expressed in the inner, hypoxic cell layers [8, 58–61]. Functionally HIF expression in both MCTS and in tumors has been linked to cell survival through the repression of pro-apoptotic signaling, repression of proliferation, and the regulation of metabolic reprograming [58, 62]. In this way, HIF signaling contributes to regionalization of MCTS cell layers and the formation of microenvironments as a function of cell distance from the MCTS surface: limited diffusion of oxygen into MCTS establishes gradients in HIF-α stabilization and subsequently tumor cell behavior. Similarly, rapid proliferation by well oxygenated cell layers and the formation of physical barriers, through tight cell-cell contacts and ECM deposition, generates gradients in glucose, catabolites, and therapeutics [32, 63]. The following sections will discuss the ways in which gradient development within the MCTS model reflects tumor cell adaptations in the avascular tumor space in vivo. These adaptations are schematized in Fig. 1.

#### Proliferation gradients within MCTS

Physiological hypoxia (5% > [O_2_] > 1%) [64] stimulates cell growth signaling through HIF-α to produce various cytokines and proteins necessary for carrying out cell division [20, 64]. Maintenance of proliferation of tumor and non-tumor cells requires an adequate supply of biomass (nucleotides, carbon sources, lipids) which is progressively diminished with decreasing oxygen levels resulting in a slowed and eventually halted replication cycle. Severe hypoxia defined as an [O_2_] < 0.13% [2] will result in replisome disassembly and exit from the cell cycle [2, 59, 65]. Spheroids larger than 400 μm in diameter develop oxygen gradients representing a range of hypoxic conditions including chronic-severe hypoxia in the spheroid core. Accordingly spheroid proliferation occurs in a regionally specified manner; there is a progressive decrease in the fraction of S-phase cells with the development of hypoxia in the MCTS core [17, 43, 46, 59, 60, 66–71]. In this way, proliferation gradients develop in a size-dependent manner.

In a recent study we found that a population of cycling cells that retain proliferative status in a hypoxic environment can be identified in MCTS larger than 500 μm in diameter formed with A673 (Ewing Sarcoma) or Lewis Lung Carcinoma cells [71]. In human tumors, identification of a similar proliferative hypoxic cell population has been negatively correlated to outcome and it is hypothesized that these cells contribute to tumor recurrence and metastasis [72–74]. The mechanisms underlying tumor cell survival and proliferation within the 3D hypoxic environment is not well studied, however the identification of such a population within the MCTS model indicates that MCTS can be used to advance our understanding of proliferation under hypoxia.

#### Hypoxia and the activation of DNA damage repair signaling

Hypoxia development drives the repression of several DDR proteins which contributes to an increased mutation rate amongst hypoxic cells [1, 4, 19, 50–52, 75]. On the other hand, numerous studies have demonstrated hypoxia-dependent activation of DDR proteins leading to stabilization of otherwise damage prone replication forks [59, 60, 76–80]. Although well described in vitro, there are still significant unknowns regarding hypoxia-induced DDR, including the clinical relevance of such signaling in vivo and the specific proteins involved in this signaling network.

There is extensive evidence showing down regulation of numerous proteins involved in homologous recombination, mismatch repair, base excision repair, and nucleotide excision repair under hypoxic conditions [1]. Homologous recombination genes RAD51 and BRCA1, and the mismatch repair protein MLH1 [53] were shown to be transcriptionally down regulated in a panel of established cell lines in response to severe hypoxia (<0.5% O_2_) [50–52]. These results were validated in vivo by the finding that both RAD51 and BRCA1 were inversely correlated to markers of hypoxia in cervical and breast cancer patients [1, 50–52]. Recent studies using MCTS reported a similar decrease in DDR proteins [9, 53]. The loss of DDR proteins correlates with a decreased ability to repair double stand breaks under oxygen deprivation [1, 19, 81, 82]. This impaired DDR response is being investigated as a potential therapeutic advantage through contextual synthetic lethality [82]. The concept proposes targeting the remaining DDR factors to induce catastrophic genomic instability. It is assumed that non-malignant cells, with an intact DDR signaling network, will be capable of repairing any therapy-induced damage. Promising results in preclinical experiments suggest that this strategy is effective when targeting PARP proteins in BRCA-deficient tumors [81, 83, 84]. This success has stimulated interest in the identification of DDR proteins operating under hypoxia as potential therapeutic targets. The ability of MCTS to recapitulate DDR protein loss validates the potential use of this model for future studies into contextual synthetic lethality.

**Fig. 2:**
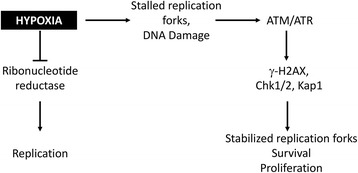
Hypoxia-induced DNA damage repair signaling. Cells attempting to proliferate under hypoxic conditions experience slowed replication due to decreased nucleotide pools [65, 85]. In the absence of oxygen, ribonucleotide reductase is impaired in its ability to produce deoxyribonucleotides required for efficient replication [65]. Replication forks stall under these conditions which can lead to DNA damage [71, 77, 78, 85]. Increased DNA damage and replication stress stimulate activation of the DNA damage repair kinases ATM and ATR [76–78]. Together these kinases phosphorylate downstream targets resulting in the stabilization of stalled replication forks and preservation of cell viability [71, 78, 85]

Compelling evidence indicates that hypoxia drives replication stress, which in turn activates the DDR kinases Ataxia Telangiectasia (ATM) and ATM-and-Rad3 related (ATR) [76, 85]. These kinases were shown to then signal through phosphorylation of downstream targets including Kap1 (S824), Chk1 (S345), Chk2 (Thr68), and H2AX (Ser139), referred to as γ-H2AX [60, 76–78]. Although ATM is activated, no DNA damage was originally reported in monolayer culture of the cell lines tested [77, 78]. However, recent studies using MCTS show increased DNA breaks under mild hypoxia using a Ewing Sarcoma cell line (A673) [71]. Together, these studies support a model where ATM and ATR activity leads to stabilization of stalled replication forks and prevention of catastrophic DNA damage, thus allowing continued cell survival and eventual restart of proliferation (Fig. 2). In vivo the consequences of hypoxia induced DDR have been difficult to study. Multiple recent papers reported correlations between γ-H2AX formation and the presence of hypoxia [86–88]. For example, in carcinomas of the uterine cervix a 1.4 fold enrichment of γ-H2AX within hypoxic tumor regions (identified through carbonic anhydrase immunoreactivity) and a 2.8 fold enrichment in severely hypoxic (pimonidazole positive) regions has been reported [88].

Studies exploring DDR in MCTS typically used γ-H2AX staining of trypsin-dissociated spheres to identify the total levels of DNA damage via flow cytometry [46, 89]. More reliable assessment of regional expression can be done through immune labeling of spheroid sections or whole mount imaging. Expression of reporter constructs located downstream of a DDR protein promotor allows for live imaging of DDR response and is of particular interest for the purposes of high throughput screening [15, 39, 45, 90]. Using these techniques, DNA damage repair has largely been explored in the context of therapeutic response to DNA damaging treatments. We recently used MCTS to describe the spatial correlation between the activation or expression of specific DDR signaling proteins, hypoxia markers, and proliferation [71]. Using immunofluorescent labeling of spheroid sections, our findings showed a strong enrichment of γ-H2AX in the hypoxic (HIF-1α, pimonidazole positive), viable cell layers of MCTS. This enrichment and the spatial proximity to necrosis was similar to in vivo studies [86–88]. Furthermore we were able to demonstrate for the first time in a 3D context that proliferating hypoxic cells activate DDR signaling which can be targeted by small molecule inhibitors. Interestingly the response to ATR inhibition differed between spheroids and monolayers, reinforcing the significant differences in cell signaling/response between 2D and 3D environments [71].

#### Altered metabolic activity in MCTS

Hypoxia within the MCTS model is tightly correlated with glucose deprivation. It has long been known that glucose starvation significantly decreases spheroid growth and viability [54, 91, 92]. Studies using radiolabeled glucose and mathematical modeling revealed a critical threshold for glucose and oxygen diffusion into the MCTS core, beyond which chronic starvation results in cell death [56, 93]. Recent findings detailing protein expression and metabolic adaptations within MCTS resemble metabolic profiles described in vivo [9, 11, 49, 54, 94, 95]. The accumulation of glycogen stores under mild hypoxia and subsequent breakdown of these stores into glucose under severe hypoxia represents one such adaptation [49]. Our studies using MCTS demonstrated regional increases in glycogen storage in the perinecrotic/hypoxic core of MCTS, correlating hypoxia development with glycogen accumulation [71]. These observations correlate well with studies showing enrichment of glycogen in perinecrotic/hypoxic zones of tumor xenografts where hypoxia altered the expression of glycogenic (GYS1) and glycolytic (PYGL) enzymes which are known to regulate MCTS glycogen storage [49]. Further glycogen accumulation within hypoxic regions of spheroids and tumor xenografts has been shown in real-time via positron emission tomography [95].

In addition to regulation of energy production, metabolic adaptations facilitate the production of reducing equivalents as a means of controlling reactive oxygen species (ROS) [96]. It is well established that hypoxia reduces oxidative phosphorylation efficiency which generates increased levels of ROS [97]. These highly reactive species oxidize lipids, proteins, and nucleic acids which disrupts cellular homeostasis and induces potentially catastrophic DNA damage [98, 99]. To counteract such effects, tumor cells upregulate metabolic pathways that generate NADPH, a powerful reducing equivalent that helps to restore glutathione levels and mitigate the negative impact of ROS [96, 98]. Some such pathways involve glucose metabolism. It was recently shown that detachment of tumor cells from the ECM disrupts glucose metabolism and induces increased ROS species [100]. In agreement with this data, the formation and growth of MCTS is dependent on sufficient glucose availability and activation of antioxidant pathways [49, 100, 101]. To facilitate growth in MCTS and within hypoxic microenvironments, tumor cells activate signaling through HIF proteins [57]. The HIF transcriptional unit is a heterodimeric complex composed of a constitutively expressed HIF-β subunit and a labile HIF-α subunit. In well oxygenated tumor regions, HIF-α proteins are degraded following proline hydroxylation by Prolyl Hydroxylase (PHD) proteins [57]. In the absence of oxygen, PHD protein function is diminished resulting in HIF-α stabilization, dimerization with HIF-β, and subsequent transcriptional regulation of numerous proteins. The majority of proteins involved in glycolysis are regulated by the HIF transcriptional complex leading to increased glycolysis under hypoxic conditions [57, 62, 102]. In addition to oxygen deprivation, ROS production impairs PHD function leading to HIF-α stabilization, further contributing to metabolic reprograming under hypoxic stress [98].

**Fig. 3:**
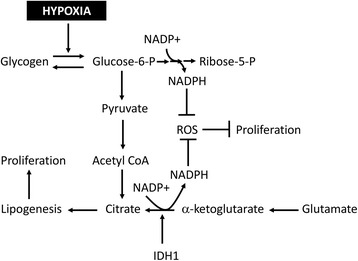
Hypoxia induces metabolic adaptations to prevent ROS related damage and maintain proliferation. Under hypoxic conditions tumor cells experience elevated levels of ROS. To mitigate the negative impact of ROS, tumor cells have been shown to increase glycogen stores which are later broken down into glucose-6-phosphate (Glucose-6-P) [49, 62]. Hypoxic cells increase flux through the pentose phosphate pathway which produces NADPH and Ribose-5-phosphate (Ribose-5-P). Ribose-5-P serves as a precursor for DNA nucleotides thus facilitating proliferation [143]. NADPH provides reducing equivalents that restore glutathione levels and neutralize ROS. NADPH is also generated through reductive carboxylation of α-ketoglutarate by Isocitrate Dehydrogenase proteins [104, 106]. Following oxygen deprivation, glutamine is metabolized to form glutamate and subsequently α-ketoglutarate [106]. The formation of citrate by IDH proteins has been shown to increase lipogenesis and facilitate proliferation [105]

HIF-α stabilization, metabolic reprograming, and ROS production have all been observed in MCTS with patterns similar to those reported in vivo [8, 22, 49, 71, 103, 104], reflecting recent reports of increased glycogen storage under hypoxic conditions facilitating continued cell proliferation and survival by increasing NADPH production [49, 62]. Similarly, metabolic reprograming under hypoxia includes increased activity from the Isocitrate Dehydrogenase proteins (IDH) which play a significant role in ROS homeostasis and proliferation under hypoxia [105–107]. IDH proteins reductively carboxylate α-ketoglutarate to form citrate and NADPH [106, 107]. This process has become increasingly appreciated as critical for cell viability under hypoxia by promoting de novo lipogenesis, restoration of cellular glutathione, and production of carbon equivalents for cell signaling [104–107]. Not surprisingly, the loss of IDH proteins leads to the suppression of MCTS growth concomitant with increased ROS production [104]. IDH appears to be involved in MCTS formation as evidenced by the inability to reproducibly form MCTS from glioma cells containing IDH mutations which alter substrate specificity [108]. Similar mutations in vivo are associated with increased patient survival due to an impaired HIF response within hypoxic tumor regions [109]. These adaptations to neutralize ROS have been schematized in Fig. 3.

The activation of glycolytic pathways is clearly replicated in MCTS as well. Numerous studies have identified increased lactic acid production within hypoxic spheroids which correlates with increased production and activity from glycolytic enzymes [94, 110, 111]. Increased glycolytic activity is indicative of an adaptive shift from oxidative phosphorylation to glycolysis as the primary source of ATP, referred to as the Warburg effect [5, 112, 113]. The ability of MCTS to mimic metabolic adaptations observed in vivo could provide a relevant model system for targeting metabolic pathways in cancer therapy.

Together these studies indicate that hypoxia development within the MCTS model generates metabolic reprograming leading to increased synthesis of glycogen, IDH reductive carboxylation, and glycolytic activity with similar distributions to that observed in vivo. Comprehensive reviews of metabolic adaptations under hypoxic conditions and the interplay of ROS with this process are available [96, 98, 114].

### MCTS in co-culture studies

The MCTS discussed thus far were composed exclusively of tumor cells. However, tumors are diverse in terms of their micro-environmental composition as well as their cellular composition. The adaptation of tumor cells to a hypoxic environment is influenced by the activity of stromal cells including endothelial cells, fibroblasts, adipocytes, macrophages, monocytes, and other cell types [115]. As in the case of tumor cells, the hypoxic environment can affect stromal cell behavior [116]. Endothelial cells are known to increase proliferation in response to a hypoxic environment and to activate a DDR signaling cascade similar to that described for tumor cells [117–119]. Accumulating evidence suggests that immune cell infiltration into the hypoxic tumor microenvironment increases angiogenesis and tumor metastasis [6, 120]. Both adipocytes and fibroblasts contribute significantly to tumor progression through the production of pro-proliferative cytokines and through production of extracellular matrix (ECM) proteins which confer chemo-resistance [6, 16, 56, 121–124].

Towards accurately recreating the tumor microenvironment in vitro, various co-culture models have been developed wherein MCTS are composed of both tumor and stromal cells. The OMS model is perhaps the most representative form of co-culture system due to the inclusion of all resident stromal cell types within the excised tissue [36, 125]. OMS have been used as a model for multiple tumor types and accurately reflect tumor growth kinetics [36, 43, 44]. In glioblastoma for example, the OMS model was found to be superior to MCTS in its ability to recreate the immuno-histochemical profile of in vivo tissues including the expression of many proteins previously correlated to hypoxia-induced aggression (CD31, CD133, P-glycoprotein, and TIMP-1) [44]. However, the inclusion of multiple cell types in the OMS model makes it difficult to determine the influence of a specific stromal cell population on the adaptive tumor cell response [36, 125].

Heterologous spheroid culture in which tumor cells are combined with one or more stromal cell types has provided unique insights into stromal and tumor cell responses to hypoxia [15, 36, 126]. The most commonly used methods of heterologous culture can be broadly described as: 1) Spheroid confrontation culture: individual spheroids are formed from stromal cells and separately from tumor cells followed by joint culture in suspension or embedded within an ECM, 2) Spheroid-monolayer culture: pre-formed MCTS are placed atop confluent monolayers, 3) Heterologous spheroid co-culture: tumor and stromal cell suspensions are combined during the sphere forming process, 4) Spheroid co-cultures in vivo: pre-formed MCTS containing stromal cells are implanted in vivo [125].

MCTS in co-culture with endothelial cells mimic several processes during hypoxia-induced angiogenesis: oxygen deprivation within the MCTS core stimulates production and accumulation of vascular endothelial growth factors (VEGF) [61, 127] which provides a stimulus for endothelial cell recruitment. In vivo, endothelial cell invasion into tumors facilitates metastasis and release of tumor cells from the growth inhibitory effects of a hypoxic environment [6]. Other MCTS co-culture models recreate a similar process and have been used to improve our understanding of the relationship between endothelial cells and tumor cells [125, 128]. For example, placement of tumor spheroids atop confluent endothelial monolayers can result in infiltration by endothelial cells expressing tumor complimentary adhesion molecules or can result in tumor-mediated destruction of endothelial vessels, reminiscent of cell death observed in vivo following vessel cooption [125, 128]. Use of confrontation or heterologous co-culture models can successfully produce vascular networks within spheroids with an increase in tumor cell viability and drug resistance [22, 129]. The invasive potential of tumor cells following vascularization is also replicated through the use of heterologous sphere culture systems [56, 122, 130]. A recent study highlighted this by showing the formation of MCTS with both endothelial and tumor cells could lead to the formation of luminal vessels in which migrating tumor cells can be seen [130], mimicking the process of tumor intravasation.

Fibroblasts are also a critical stromal cell population that contribute to tumor progression through the release of proliferative and pro-metastatic growth factors and foster the creation of a drug resistant environment by depositing ECM which hinders drug diffusion [122, 125, 126]. Each of these characteristics are replicated in heterologous sphere culture [16, 56, 122, 124–126]. Within a pro-angiogenic environment, fibroblasts take on a mural cell phenotype and augment angiogenesis through the release of VEGF, matrix metalloproteinases (MMP), and other growth factors [16, 56, 122, 124, 126]. In vitro this process can be studied through the “mini-tumor model” in which endothelial cells, fibroblasts, and tumor cells are co-cultured in 3D [122]. This tri-culture model replicated pathological formation of luminal vascular structures bordered by fibroblasts and tumor cells which are both dependent on angiogenic growth factors and responsive to anti-angiogenic therapeutics [122]. This mini-tumor model has significant potential for studying the refractory response of tumor and stromal cells following anti-angiogenic therapy and the specific mechanisms used to circumvent these treatments.

Immune cells are a major regulatory factor during tumor progression [5]. Within the hypoxic tumor environment, there are indications that immune cells facilitate tumor aggression through increased angiogenesis and immunosuppression [115, 131]. In vivo immune cells aggregate within hypoxic, peri-necrotic regions [131]. These observations have been mirrored in MCTS co-cultures and indicate potential utility for MCTS as an investigational tool to replicate in vivo conditions [56, 132]. In support of this, immune cell influence over angiogenesis has been described by a series of studies through the inclusion of monocytes in co-culture with tumor spheroids to show immune-dependent increases in production of VEGF, ECM degrading proteins, and in vivo angiogenesis through MCTS co-culture in skinfold window chambers [56, 131–133]. A particular advantage to MCTS co-culture in modelling the hypoxic microenvironment is the accumulation of immunomodulatory metabolites and cytokines.

Levels of lactic acid are commonly high in hypoxic tumor regions due to increased glycolytic activity and HIF mediated production of lactic acid transporters [134]. In vitro studies show that hypoxia-induced increases in lactic acid and VEGF can decrease dendritic cell differentiation and maturation while increasing monocyte conversion to M2 macrophages, ultimately resulting in immunosuppression [56, 131, 135–137]. The MCTS co-culture models are well suited to model a hypoxic microenvironment with high metabolite and growth factor levels. Hypoxic spheroids show higher levels of lactic acid which correlates with decreased immune cell invasion, reduced production of colony stimulating factor [131, 136] and decreased dendritic cell maturation in hypoxic conditions [131, 138].

### MCTS as a model to determine therapeutic efficacy in heterogeneous environments

Aside from instigating an aggressive phenotype, tumor hypoxia presents several challenges to therapeutic intervention [3, 139]. Hypoxia has been appreciated for its ability to reduce radiation effectiveness for many decades [24, 84, 140]. Since this phenomenon was described, a significant amount of evidence has amassed demonstrating the ways a hypoxic environment renders tumor cells privileged, or resistant, to multiple therapeutic modalities [3, 13, 22, 23, 44, 63, 88, 139, 141, 142]. Several studies have detailed the contribution of stromal cells to chemo-resistance in the MCTS model [14, 16, 122, 124] and technological advances are allowing more complex co-culture systems to be developed [15, 45]. The use of MCTS for screening of therapeutics has been extensively reviewed elsewhere [39].

## Conclusions

The negative impact of hypoxia on tumor prognosis warrants a significant effort to better understand and target hypoxic tumor cell adaptations. The MCTS model is an established technique with untapped potential to improve our understanding of subpopulations within a tumor. With growing therapeutic interest in targeting metabolic pathways, DDR proteins, and contextual synthetic lethality, the MCTS model could be used to great effect.

## Abbreviations

2D: Two dimensional cell culture monolayer culture
3D: Three dimensional
A673: Human Ewing Sarcoma cell line (CRL-1598; ATCC)
ATM: Ataxia Telangiectasia
ATR: ATM and Rad3-related
BRCA1: Breast Cancer 1
CD133: Prominin-1
CD31: Platelet Endothelial Cell Adhesion Molecule
Chk1/2: Checkpoint kinase 1 and 2
DDR: DNA damage repair
ECM: Extracellular matrix
GYS1: Glycogen synthase 1
H2AX: Histone variant 2A.X
HIF-α: Hypoxia Inducible Factor α subunit
IDH: Isocitrate Dehydrogenase
Kap1: KRAB-associated protein-1
MCTS: Multicellular Tumor Spheroids
MLH1: MutL homolog 1
MMP: Matrix metallopeptidase
OMS: Organotypic Multicellular Sphere
PARP: Poly (ADP-ribose) polymerase
PYGL: Glycogen phosphorylase L (Liver)
RAD51: RAD51 recombinase
TDTS: Tumor Derived Tumor Sphere
TIMP-1: TIMP metallopeptidase inhibitor 1
TS: Tumor Sphere
VEGF: Vascular Endothelial Growth Factor
γ-H2AX: H2AX phosphorylated on serine-139
